# Enhanced Hydrogen Storage Properties of MgH_2_ Using a Ni and TiO_2_ Co-Doped Reduced Graphene Oxide Nanocomposite as a Catalyst

**DOI:** 10.3389/fchem.2020.00207

**Published:** 2020-03-24

**Authors:** Liang Zeng, Peilin Qing, Fangfang Cai, Xiantun Huang, Haizhen Liu, Zhiqiang Lan, Jin Guo

**Affiliations:** ^1^School of Physical Science and Technology, Guangxi Novel Battery Materials Research Center of Engineering Technology, Guangxi Colleges and Universities, Key Laboratory of Novel Energy Materials and Related Technology, Guangxi University, Nanning, China; ^2^Guangxi Colleges and Universities Key Laboratory of Structure Research and Performance Development of Rare Earth Alloy, School of Materials Science and Engineering, Baise University, Baise, China

**Keywords:** magnesium, reduced graphene oxide, hydrogen storage properties, apparent activation energy, nanocomposites

## Abstract

To improve the hydrogen storage properties of Mg/MgH_2_, a Ni and TiO_2_ co-doped reduced graphene oxide [(Ni-TiO_2_)@rGO] nanocomposite is synthesized by a facile impregnation method and introduced into Mg via ball milling. The results demonstrated that the dispersive distribution of Ni and TiO_2_ with a particle size of 20–200 nm in the reduced graphene oxide matrix led to superior catalytic effects on the hydrogen storage properties of Mg-(Ni-TiO_2_)@rGO. The initial hydrogenation/dehydrogenation temperature for Mg-(Ni-TiO_2_)@rGO decreased to 323/479 K, 75/84 K lower than that of the additive-free sample. The hydrogen desorption capacity of the Mg-(Ni-TiO_2_)@rGO composite released 1.47 wt.% within 120 min at 498 K. When the temperature was increased to 523 K, the hydrogen desorption capacity increased to 4.30 wt.% within 30 min. A hydrogenation/dehydrogenation apparent activation energy of 47.0/99.3 kJ·mol^−1^ was obtained for the Mg-(Ni-TiO_2_)@rGO composite. The improvement in hydrogenation and dehydrogenation for the Mg-(Ni-TiO_2_)@rGO composite was due to the reduction of the apparent activation energy by the catalytic action of (Ni-TiO_2_)@rGO.

## Introduction

Hydrogen is a highly promising clean energy source. Hydrogen storage materials represent one of the key technologies for the development of hydrogen energy. Magnesium is considered to be the most promising hydrogen storage material because of its high theoretical hydrogen storage capacity (7.6 wt.%), abundant reserves and low cost (Jain et al., 2010; Liu et al., 2015). However, its practical application is limited due to its thermodynamic stability and poor kinetic performance. To overcome these weaknesses, methods such as alloying (Oh et al., 2016; Hardian et al., 2018; Li et al., 2018), nanocrystallization (Wagemans et al., 2005; Li et al., 2007) and the addition of catalysts has been employed (Cui et al., 2014; Huang et al., 2017; Zhang et al., 2017; Wang et al., 2019; Yang et al., 2019).

The addition of catalysts is one of the most effective ways to improve the hydrogen storage performance of Mg-based alloys. Cui et al. (2014) proved that the hydrogen storage properties of Mg can be improved by adding Ti, Nb, V, Co, Mo, and Ni metals. Wang et al. (2019) introduced a two-dimensional layered NbTiC solid solution MXene to MgH_2_ and observed that the initial dehydrogenation temperature of the MgH_2_-9 wt.% NbTiC sample was reduced to 468 K, which is 80 K lower than that of MgH_2_. These improvements were attributed to the breakage of Mg-H bonds and the detachment of H_2_ from the NbTi surface. Liu et al. (2016) reported a MgH_2_-5 wt.% Ti_3_C_2_ sample that began to release hydrogen at 458 K and can uptake ~6.1 wt.% H_2_ within 30 s at 423 K. Chen et al. (2019) prepared highly dispersed Co metal nanoparticles on TiO_2_ via a solvothermal method and found that the combined catalytic action of Ti and Co on the hydrogen de/absorption properties of MgH_2_ was more effective than that of TiO_2_ or Co alone. The desorption peak temperature for the MgH_2_-Co/TiO_2_ composite was 526.2 K, which was 53.2, 94.2, and 132.2 K lower than that of MgH_2_-TiO_2_ (561.4 K), MgH_2_-Co (602.4 K) and ball-milled MgH_2_ (640.4 K), respectively.

Among the various catalysts, nickel is considered to be the most effective to improve the hydrogen storage properties of magnesium hydride (MgH_2_). MgH_2_ nanoparticles doped with Ni were reported by Xie et al. (2009), who reported that 6.1 wt.% of hydrogen can be released from Mg-10 wt.% Ni hydride within 10 min at 523 K. Ma et al. (2018) proposed a method to dope MgH_2_ with carbon-supported nano Ni and found that the kinetics of MgH_2_ can be significant improved. The Mg-Ni@C composite can release hydrogen thoroughly within 500 s at 573 K, which was 3,000 s faster than that of the additive-free sample. It has been proven in our previous studies that reduced graphene oxide-supported nickel acting as a catalyst can effectively improve the comprehensive hydrogen storage performance of MgH_2_ and the interaction between graphene and nickel is more effective than that of graphene or nickel separately (Lan et al., 2019). As described above, the comprehensive hydrogen storage performance of MgH_2_ can be improved effectively under the synergetic catalytic action of the multiple catalysts working together. In addition, theoretical calculations have also proved that the combined action of catalysts is more effective than that of a single catalyst (Zhou et al., 2014; Sun et al., 2016).

In this work, we synthesized a Ni and TiO_2_ co-doped reduced graphene oxide [(Ni-TiO_2_)@rGO] nanocomposite via a chemical reduction method and introduced it into nano Mg by mechanical alloying. We then studied the effect of the (Ni-TiO_2_)@rGO nanocomposite on the hydrogen storage properties of MgH_2_ by SEM, TEM, and DSC.

## Methods

### Synthesis of Mg Nanoparticles

First, 12.818 g of naphthalene and 0.694 g of lithium were added into tetrahydrofuran and then stirred for 12 h at room temperature to obtain a naphthalene lithium solution. Meanwhile, 3.81 g of MgCl_2_ was added to 400 mL of tetrahydrofuran and stirred until MgCl_2_ was completely dissolved. Subsequently, the obtained MgCl_2_ solution was added to the naphthalene lithium solution drop by drop and stirred for 3 h to ensure that Mg^2+^ was reduced to Mg. After centrifugal washing three to five times, the Mg products were dewatered by a vacuum pump for 4 h at room temperature. The dehydrated samples were then vacuum pumped for 10 h at 343 K to obtain Mg powder.

### Preparation of (Ni-TiO_2_)@rGO Composite

**Figure 1:**
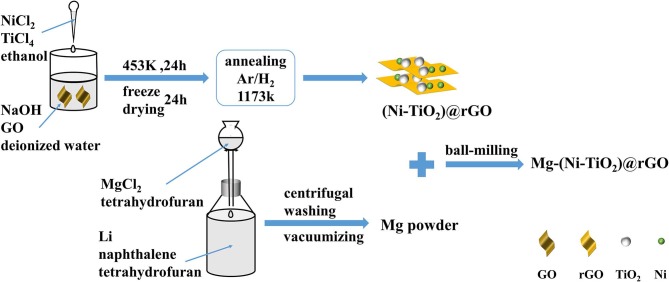
Schematic preparation of (Ni-TiO_2_)@rGO composite.

The preparation scheme of the (Ni-TiO_2_)@rGO nanocomposite is shown in Figure 1. To start, 1.5 g of sodium hydroxide and 200 mg of graphite oxide were added to 200 mL of deionized water, respectively, and then dispersed and stirred for 1 h by ultrasound. Next, 5.5 mL of TiCl_4_ liquid were injected into 100 mL of ethanol. After stirring for 1 h, 10 mL of the obtained solution and 0.884 g of NiCl_2_ were added into 100 mL of ethyl alcohol and stirred for 1 h. The mixture was injected into a sodium hydroxide solution and stirred for 12 h. The samples were heated to 453 K and held for 24 h in the reactor, and then washed in deionized water and dried for 24 h in a lyophilizer. The dried samples were heated for 3 h at 1,173 K in a sintering furnace under a mixed gas (Ar:H_2_ = 93:7) atmosphere and then cooled with the furnace. The reduced graphene oxide supported Ni (Ni@rGO) and TiO_2_ (TiO_2_@rGO) were prepared by similar methods.

### Preparation of Mg-(Ni-TiO_2_)@rGO Composite

The as-prepared Mg powers were divided into four parts, three of them mixed with 10 wt.% Ni@rGO, 10 wt.% TiO_2_@rGO and 10 wt.% (Ni-TiO_2_)@rGO, respectively. Afterwards, all the samples were milled for 1 h with a milling speed of 500 rpm and a ball-to-powder weight ratio of 40:1 under Ar atmosphere protection, yielding composites denoted as Mg, Mg-Ni@rGO, Mg-TiO_2_@rGO, and Mg-(Ni-TiO_2_)@rGO, respectively.

### Characterization

Microstructural characterizations of the samples were performed via X-ray diffraction (XRD; Miniflex 600, Rinku; Cu-Kα radiation, 40 kV and 200 mA), field emission scanning electron microscopy (FE-SEM; SU8020, HITACHI) and transmission electron microscopy (TEM; FEI Tecnai G2, f20 s-twin 200 kV). The distributions of Ni, Ti, O, and C elements were observed using energy-dispersive X-ray spectrometry (EDS) in conjunction with SEM. The hydrogenation and dehydrogenation were performed on a Sievert-type device. After they were fully activated, the samples were heated from room temperature to 653 K with a rate of 1 K·min^−1^ under a hydrogen pressure of 6 MPa during hydrogenation and with a rate of 0.5 K·min^−1^ under a hydrogen pressure of 0.01 MPa during dehydrogenation. The hydrogenation kinetics were determined at 323, 348, 373, and 398 K, as were the isothermal behaviors of hydrogenation. The isothermal behaviors of dehydrogenation were measured at 498, 523, 548, and 573 K.

## Results and Discussion

**Figure 2:**
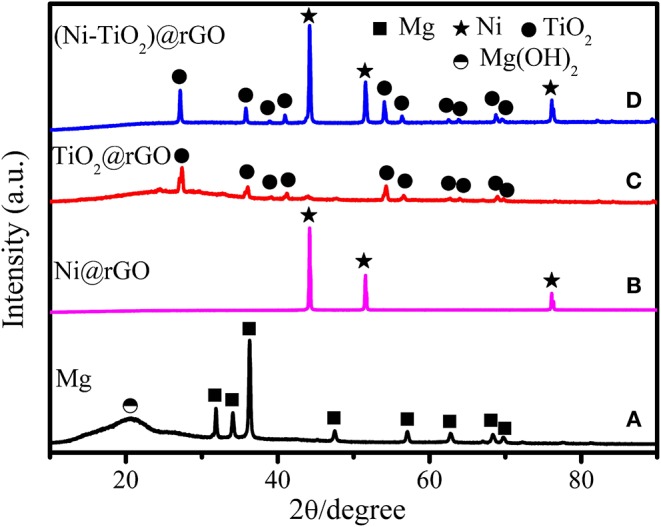
XRD patterns of as-prepared **(A)** Mg, **(B)** Ni@rGO, **(C)** TiO_2_@rGO, and **(D)** (Ni-TiO_2_)@rGO nanocomposites.

**Figure 3:**
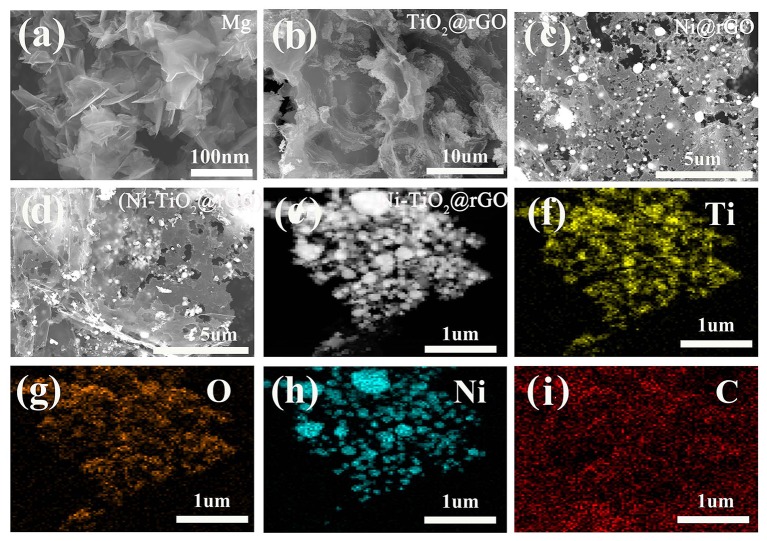
FE-SEM images for **(a)** Mg, **(b)** TiO_2_@rGO, **(c)** Ni@rGO and **(d,e)** (Ni-TiO_2_)@rGO, and EDS images for **(f)** Ti, **(g)** O, **(h)** Ni, and **(i)** C elements.

**Figure 4:**
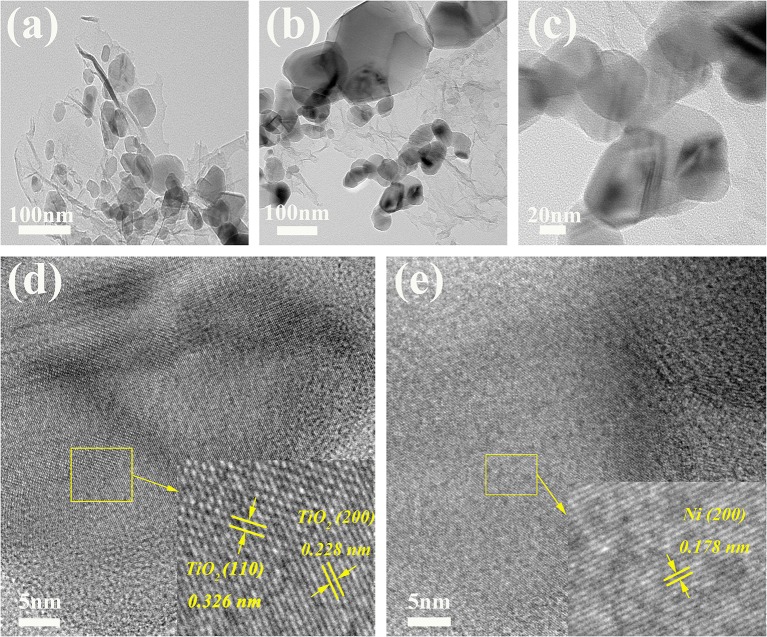
TEM **(a–c)** and HRTEM **(d,e)** images of (Ni-TiO_2_)@rGO nanocomposites.

Figure 2 shows the XRD patterns of the as-prepared Mg, Ni@rGO, TiO_2_@rGO, and (Ni-TiO_2_)@rGO nanocomposites. As shown, the diffraction peaks appear at 31.9°, 34.1°, 36.3°, 47.5°, 57.1°, 62.7°, 67.0°, 69.7°, 72.3° and 77.7° in Figure 2A corresponding to the (100), (002), (101), (102), (110), (103), (200), (112), (201), (004), and (202) planes of Mg phase with rutile structure (Standard JCPDS card 00-035-0821), respectively. A diffraction peak at about 2θ=20.7° corresponds to Mg(OH)_2_ phase which may be caused by the hydrolysis of Mg in the glovebox or during the milling process (Song et al., 2010). Besides, no other phases are found in Figure 2A, indicating that Mg has been well-prepared via chemical reduction method in aqueous solution. And three diffraction peaks appear at 44.2°, 51.6°, and 76.1° in Figure 2B corresponding to the (111), (200), and (220) planes of Ni fcc phase (Standard JCPDS card 04-0850), respectively. Additionally, the characteristic diffraction peaks of the TiO_2_ phase occur at 27.4°, 36.1°, 39.2°, 41.2°, 54.3°, 56.6°, 62.7°, 64.0°, 69.0°, and 69.8° (Figures 2C,D), arising from the crystal plane indices of (110), (101), (200), (111), (211), (220), (002), (310), (301), and (112) (JCPDS card 21-1276), respectively. Figure 3 shows the FE-SEM images for (a) Mg, (b) TiO_2_@rGO, (c) Ni@rGO, and (d,e) (Ni-TiO_2_)@rGO and EDS images for (f) Ti, (g) O, (h) Ni, and (i) C elements. Nanosheets structure with a size of about 100 nm for as-prepared Mg can be observed in Figure 3a. It also can be found from Figures 3b–d that numerous nanosized particles are homogenous dispersion on the surface of the sheets. The sheet structure materials should be correspond to the reduced graphene oxide. To investigate the presence of the elements and the distribution of the particles, SEM-EDS characterization was employed for the (Ni-TiO_2_)@rGO composite (see Figures 3e–i). It can be observed that Ti, O and Ni elements are well-scattered on the surface of the reduced graphene oxide sheets. Based on the XRD results in Figure 2D, it can be deduced that the (Ni-TiO_2_)@rGO nanocomposites have been fabricated successfully via a facile impregnation method. To further investigate the microstructure of the as-prepared sample, TEM and HRTEM were applied to the (Ni-TiO_2_)@rGO nanocomposite and the results are shown in Figure 4. Figure 4 shows the TEM (Figures 4a–c) and HRTEM (Figures 4d–e) images of (Ni-TiO_2_)@rGO nanocomposites. The HRTEM images indicated that numerous particles with a size of 20–200 nm were anchored on the rGO surface. Additionally, lattice spacing of 0.326 and 0.228 nm, corresponding to the (110) and (200) planes of TiO_2_, could be observed in Figure 4d. A lattice spacing of 0.178 nm matching with the (200) plane of Ni could also be detected in Figure 4e, further indicating that the (Ni-TiO_2_)@rGO nanocomposite had been successfully fabricated via a facile impregnation method.

**Figure 5:**
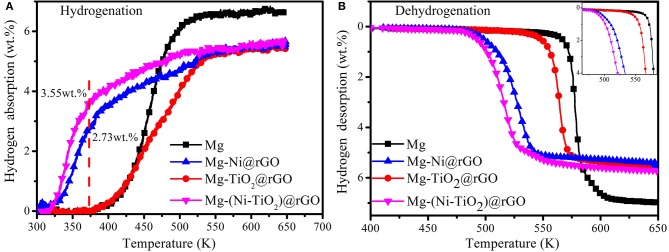
Hydrogenation **(A)** and dehydrogenation **(B)** curves of Mg and Mg-Ni@rGO, Mg-TiO_2_@rGO, and Mg-(Ni-TiO_2_)@rGO composites.

**Figure 6:**
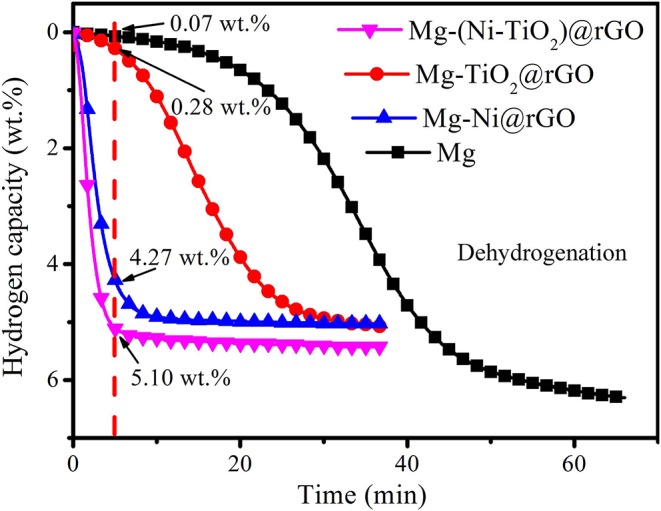
Dehydrogenation curves of Mg and Mg-Ni@rGO, Mg-TiO_2_@rGO, and Mg-(Ni-TiO_2_)@rGO composites performed at 573 K.

Figure 5 shows the hydrogenation and dehydrogenation curves of Mg and the Mg-Ni@rGO, Mg-TiO_2_@rGO and Mg-(Ni-TiO_2_)@rGO composites. As shown in Figure 5A, the Ni@rGO and (Ni-TiO_2_)@rGO modified samples could uptake hydrogen at ~323 K and the two samples absorbed hydrogen quickly in the temperature range of 323–373 K. A total of 2.73 wt.% H_2_ was obtained in the Ni@rGO modified sample when heated to 373 K at a temperature rate of 1 K·min^−1^. While for the Ni and TiO_2_ co-doped rGO modified sample [(Ni-TiO_2_)@rGO], the hydrogen absorption capacity increased to 3.55 wt.% under the same conditions, indicating that the catalytic effect of (Ni-TiO_2_)@rGO on the hydrogen absorption of Mg was more effective than that of Ni@rGO. Compared with the (Ni-TiO_2_)@rGO and Ni@rGO nanocomposites, there was almost no hydrogen uptaken in Mg and Mg-TiO_2_@rGO until the temperature reaches ~375 K. During the dehydrogenation process, the dehydrogenation temperature was significantly decreased under the catalytic action of TiO_2_@rGO, Ni@rGO and (Ni-TiO_2_)@rGO (Figure 5B). For example, the initial dehydrogenation temperature for Mg was ~563 K and this temperature reduced to 545 K when decorated by the TiO_2_@rGO nanocomposite, which was ~18 K lower than that of the Mg sample. With Ni@rGO and (Ni-TiO_2_)@rGO added, the initial dehydrogenation temperatures further decreased to 485 and 479 K, respectively. These results suggested that the Ni@rGO and (Ni-TiO_2_)@rGO nanocomposites acted as catalysts to improve the hydrogen absorption/desorption properties of Mg. In particular, the Mg-(Ni-TiO_2_)@rGO composite exhibited excellent hydrogen absorption/desorption properties compared to the other samples. It is deduced that Ni and TiO_2_ particles embedded in the rGO matrix or dispersed on the surface of the rGO sheets may have a positive effect on the hydrogen storage properties of MgH_2_. Theoretical prediction suggested that the addition of TM to MgH_2_ could weaken the bond between Mg and H, which made the doped TM-MgH_2_ systems unstable and leaded to the decreasing of their desorption temperatures (El Khatabi et al., 2016). Zhang et al. (2015) reported that the dehydrogenation enthalpy and the dehydrogenation activation energy of MgH_2_ can be decreased by doping with grapheme. Doping MgH_2_ with TiO_2_@C composites can elongate the lengths and weaken the strengths for Mg-H bond, resulting in the decrease of the hydrogenation/dehydrogenation temperature of the MgH_2_-TiO_2_@C system (Zhang et al., 2018). As mentioned above, the combined action of Ni, TiO_2_, and rGO on Mg/MgH_2_ should enhance the hydrogenation/dehydrogenation performances effectively. To investigate the effect of TiO_2_@rGO, Ni@rGO and (Ni-TiO_2_)@rGO on the dehydrogenation kinetics, the dehydrogenation curves of the samples were obtained at 573 K and are shown in Figure 6. The hydrogen desorption capacities of Mg, Mg-TiO_2_@rGO, Mg-Ni@rGO, and Mg-(Ni-TiO_2_)@rGO were 6.31, 5.07, 5.04, and 5.43 wt.%, respectively. Although the hydrogen storage capacity decreased somewhat, the addition of TiO_2_@rGO, Ni@rGO, and (Ni-TiO_2_)@rGO enhanced the kinetic properties of dehydrogenation. As shown in Figure 6, Mg-(Ni-TiO_2_)@rGO, Mg-Ni@rGO, Mg-TiO_2_@rGO, and Mg released 5.10, 4.27, and 0.28 wt.% of H_2_ within 5 min, accounting for 93.9, 84.7, and 5.5% of the reversible hydrogen storage capacity, respectively. These results suggest that the dehydrogenation kinetic property of Mg can be enhanced by adding (Ni-TiO_2_)@rGO nanocomposites, which is superior to that of Co@C nanoflowers (Li et al., 2017), ZrCo nanosheets (Zhang et al., 2020). Additionally, almost no hydrogen was released from the Mg sample at 573 K. Although the catalytic effect of TiO_2_@rGO is poor, the synergistic effect of Ni and TiO_2_ co-doped reduced graphene oxide significantly enhanced the dehydrogenation kinetics for MgH_2_.

**Figure 7:**
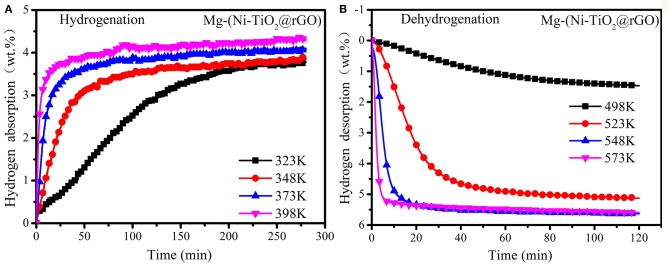
Hydrogenation/dehydrogenation curves of Mg-(Ni-TiO_2_)@rGO composite at different temperatures. **(A)** Hydrogenation **(B)** Dehydrogenation.

**Figure 8:**
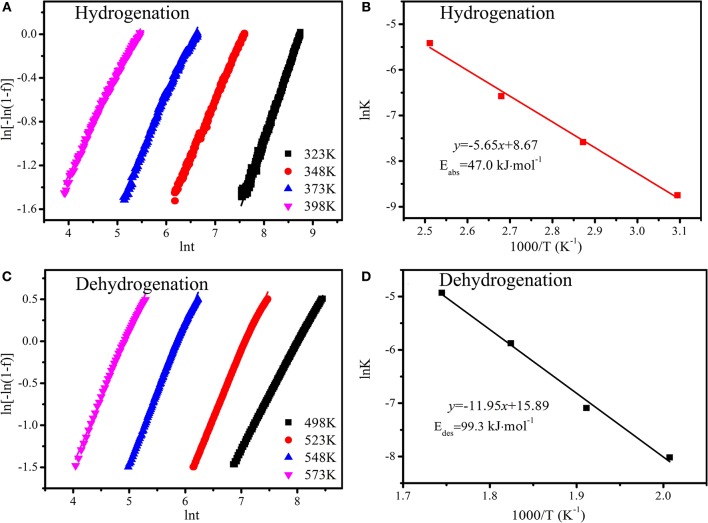
Plot of ln[-ln(1-*f* (*t*))] vs. ln*t* curves **(A,C)** and Arrhenius plots **(B,D)** for the Mg-(Ni-TiO_2_)@rGO composite: **(A,B)** hydrogenation; **(C,D)** dehydrogenation.

To further study the Ni and TiO_2_ co-doped reduced graphene oxide effect on the hydrogen storage properties of Mg, isothermal absorption/desorption curves, performed at 323, 348, 373, and 398 K, were plotted in Figure 7. As shown in Figure 7A, the hydrogen absorption capacity of the Mg-(Ni-TiO_2_)@rGO composite reached 4.0 wt.% within 60 min at 398 K. Even at 323 K, the Mg-(Ni-TiO_2_)@rGO composite could uptake 3.61 wt.% of H_2_ within 200 min. At the initial stage, the curves are relatively steep, which corresponds to the rapid hydrogen absorption process. And then the curves become flat as the hydrogen absorption time increasing. The hydrogen absorption capacity increases as the temperature rise. A hydrogen capacity of 3.76, 3.82, 4.06, and 4.32 wt.% is obtained when the isothermal test temperature is 323, 348, 373, and 398 K, respectively. The dehydrogenation kinetics of the Mg-(Ni-TiO_2_)@rGO composite carried out at 498, 523, 548, and 593 K are shown in Figure 7B. The hydrogen desorption capacity of the Mg-(Ni-TiO_2_)@rGO composite released 1.47 wt.% within 120 min at 498 K. When the temperature increases to 523 K, the hydrogen desorption capacity increased to 4.30 wt.% within 30 min. Additionally, when the hydrogen desorption temperature further increased to 548 and 573 K, with 5.0 wt.% of H_2_ released in only 11.1 and 4.4 min, respectively. These results suggested that the hydrogenation/dehydrogenation kinetics for Mg could be enhanced significantly by the addition of the (Ni-TiO_2_)/rGO composite, which was better than Mg nanocrystals (Norberg et al., 2011). To investigate the catalytic action of the addition of (Ni-TiO_2_)/rGO on the hydrogenation/dehydrogenation of MgH_2_, the apparent activation energy (E_a_) is employed to the hydrogenation/dehydrogenation process, which is calculated by using the Johnson–Mehl–Avrami (JMA) equation. For the JMA model, it can be described as follows (Srinivas et al., 2008; Muthukumar et al., 2009):

(1) $$\begin{array}{l} ln\left[-\ln\left(1-f\left(t\right)\right)\right]=\eta lnk+\eta lnt \end{array}$$

where *f(t)*, η and *k* represent the time-dependent reacted fraction, reaction order and effective kinetic parameter, respectively. Based on the hydrogenation/dehydrogenation kinetics curves at different temperatures (Figure 7), a linear relation curve between ln[-ln(1-*f* (*t*))] and lnt can be plotted in Figures 8A,C. The slope value (*k*) of the linear line can be obtained from η and ηln*k* from the ln[-ln(1-*f* (*t*))] vs. ln*t* fitting. Thus, the apparent activation energies (E_a_) of the hydrogenation/dehydrogenation process are obtained by using Arrhenius equation:

(2) $$\begin{array}{l} lnk=-\frac{E_{a}}{RT}+\;lnk_{0} \end{array}$$

where *E*_*a*_, *k*_0_, *R* and *T* represent the apparent activation energy, Arrhenius pre-exponential factor, gas constant (8.31 J·mol^−1^·K^−1^) and absolute temperature (K), respectively. The Arrhenius plots for hydrogenation and dehydrogenation of the sample are shown in Figures 8B,D. The apparent activation energy can be calculated by using the slope of the curve. The calculated hydrogenation E_a_ value of the Mg-(Ni-TiO_2_)@rGO composite was 47.0 kJ·mol^−1^, significantly lower than that of Mg nanocrystals (115–122 kJ·mol^−1^) (Norberg et al., 2011). Usually, hydrogen atoms first adsorb on the alloy surface forming new nuclei, then the new nuclei grow up until the hydride covers the metal particle surface and finally the hydrogen atoms diffuse into the alloy body (Takeichi et al., 2014). Thus, during the hydrogenation process, the hydrogen atoms need to overcome an energy barrier to diffuse into the alloy bulks. In comparison to pure Mg, when modified by the (Ni-TiO_2_)@rGO composite, the apparent activation energy for hydrogen absorption in Mg decreases dramatically. The decrease of the energy barrier is conducive to hydrogen atom diffusion in bulk Mg. For dehydrogenation, the E_a_ of the Mg-(Ni-TiO_2_)@rGO composite, 99.3 kJ·mol^−1^, can also be obtained from Figure 8D. The dehydrogenation apparent activation energy is far lower than that of Mg nanocrystals (126–160 kJ·mol^−1^) (Norberg et al., 2011). Therefore, the improvement in hydrogenation and dehydrogenation for the Mg-(Ni-TiO_2_)@rGO composite was due to the reduction of the apparent activation energy by the catalytic action of (Ni-TiO_2_)@rGO.

## Conclusion

In this work, Ni@rGO, TiO_2_@rGO and (Ni-TiO_2_)@rGO nanocomposites were prepared via a facile impregnation method and introduced into Mg via ball milling. The microstructure of the nanocomposites was studied by XRD, SEM and TEM. The Ni@rGO, TiO_2_@rGO, and (Ni-TiO_2_)@rGO nanocomposite effects on the hydrogen storage properties of Mg were investigated. The integrated hydrogen storage properties of Mg were enhanced by decorating with the Ni@rGO, TiO_2_@rGO, and (Ni-TiO_2_)@rGO nanocomposites. In particular, the catalytic effect of (Ni-TiO_2_)@rGO nanocomposites on the hydrogen storage properties was more effective than that of Ni@rGO or TiO_2_@rGO. For example, with a heating rate of 1 K·min^−1^, 2.73 wt.% H_2_ can be obtained for the Ni@rGO modified sample when heated to 373 K. While for the Ni and TiO_2_ co-doped rGO modified sample, the hydrogen storage capacity increases to 3.55 wt.% under the same conditions, which was 1.3 times that of the Ni@rGO modified sample. However, almost no hydrogen was uptaken in the pure Mg and TiO_2_@rGO-modified samples. The addition of (Ni-TiO_2_)@rGO significantly reduced the apparent activation energy of hydrogenation and dehydrogenation for Mg, which resulted in enhanced hydriding kinetic properties for Mg.

## Data Availability Statement

All datasets generated for this study are included in the article/supplementary material.

## Author Contributions

LZ and PQ carried out the experimental work and the data collection and interpretation. FC, XH, and HL participated in the design and coordination of experimental work, and acquisition of data. JG and PQ participated in the design and analyzed the data of the manuscript. LZ carried out the study design, the analysis and interpretation of data and drafted the manuscript. All authors read and approved the final manuscript.

### Conflict of Interest

The authors declare that the research was conducted in the absence of any commercial or financial relationships that could be construed as a potential conflict of interest.
